# Efficacy of MRI and clinical findings of Lidocaine injection combined with manual therapy in frozen shoulder—A prospective, randomized, single-blinded, sham-controlled trial

**DOI:** 10.1371/journal.pone.0328783

**Published:** 2025-08-06

**Authors:** Gopal Nambi, Mshari Alghadier, Elturabi Elsayed Ebrahim, Mudathir Mohamedahmed Eltayeb, Dena Eltabey Sobeh, Osama R. Aldhafian, Shahul Hameed Pakkir Mohamed, Naif Nwihadh Alshahrani, Faizan Z. Kashoo, Hariraja Muthusamy, Radhakrishnan Unnikrishnan, Alaa Jameel A. Albarakati

**Affiliations:** 1 Department of Health and Rehabilitation Sciences, College of Applied Medical Sciences, Prince Sattam bin Abdulaziz University, Al Kharj, Saudi Arabia; 2 Department of Medical & Surgical Nursing, College of Nursing, Prince Sattam bin Abdulaziz University, Al Kharj, Saudi Arabia; 3 Department of Surgery, College of Medicine, Prince Sattam bin Abdulaziz University, Al Kharj, Saudi Arabia; 4 Department of Physical therapy, Faculty of Applied Medical Sciences, University of Tabuk, Tabuk, Saudi Arabia; 5 Saveetha College of Physiotherapy, Saveetha Institute of Medical and Technical Sciences, (Deemed to the University), Chennai, TamilNadu, India; 6 Orthopedic Surgery Department, King Fahad Medical City, Ministry of Health, Riyadh, Saudi Arabia; 7 Department of Physical Therapy and Health Rehabilitation, College of Applied Medical Sciences, Majmaah University, Majmaah, Saudi Arabia; 8 Department of Surgery, College of Medicine, Umm Al-Qura University, Al-Qunfudah Branch, Makkah, Saudi Arabia; AIIMS: All India Institute of Medical Sciences, INDIA

## Abstract

**Background:**

Frozen shoulder, or adhesive capsulitis, is a debilitating condition characterized by progressive pain and restricted range of motion in the glenohumeral joint. A wide range of interventions has been explored for its management, including conservative physiotherapy approaches such as thermotherapy, manual therapy, and therapeutic exercises. Pharmacological interventions, including pain medications, and muscle relaxants are commonly employed to alleviate symptoms. Additionally, intra-articular corticosteroid injections have been shown to provide short-term relief. While each modality offers potential benefits, the optimal treatment strategy remains a subject of ongoing investigation.

**Purpose:**

The objective of this study is to investigate the clinical and magnetic resonance image (MRI) changes after lidocaine injection with manual therapy in frozen shoulder.

**Study design/ setting:**

Randomized, single-blinded controlled study conducted at University hospital.

**Patient sample:**

Sixty eligible participants were divided into an active group (n = 30; Lidocaine injection with active manual therapy) and the placebo group (n = 30; Lidocaine injection with placebo manual therapy) 4 sessions per week for 4 weeks.

**Outcome measures:**

The primary outcome was pain intensity, measured with the visual analogue scale and the other outcome measures were range of motion (ROM), functional disability, thickness of corocohumeral ligament (CHL) by MRI, and quality of life which was measured at baseline, after 4 weeks, 8 weeks and at 6 months.

**Results:**

The VAS score at 4 weeks shows an improvement 2.4 (CI 95% 2.08 to 2.71) in the active group than the placebo group. Similar effects have been noted after 8 weeks 3.0 (CI95% 2.68 to 3.31) and at 6 months 2.5 (CI95% 2.18 to 2.81). Similar statistically significant improvements were found in the ROM (abduction & Lateral rotation), functional disability, thickness of CHL ligament, status and quality of life (p = 0.001).

**Conclusion:**

Lidocaine injection with active manual therapy consists of scapula mobilization and posterior capsular stretching was superior to placebo group for improving pain, ROM, functional disability, and quality of life in people with frozen shoulder.

**Trial registration:**

ClinicalTrials.gov CTRI/2020/04/024853

**Contribution of the paper:**

- This study found the differences in effects between Lidocaine injection with active and placebo manual therapy in frozen shoulder.
- The study provided a new evidence for the selection of right therapeutic intervention for frozen shoulder in clinical practice.
- These reports helpful for the physical therapists to prevent and treat the symptoms and consequences of frozen shoulder.

## 1. Introduction

According to the American Orthopedic Association (AOA), adhesive capsulitis, commonly referred to as frozen shoulder (FS), is a prevalent orthopedic condition affecting the shoulder joint, marked by a gradual onset of pain and restricted movement [1]. The condition typically follows a pattern of capsular restriction, with external rotation being the most affected movement, followed by abduction and flexion, and internal rotation being the least affected. Passive movements beyond the restricted ranges are often intensely painful, leading to significant limitations in daily activities. FS affects approximately 3% to 5% of the population [2]. Despite extensive research, the exact cause and mechanism of the condition remain unclear [2,3].

The clinical stages of frozen shoulder are classified as the painful stage, stiffening stage, and thawing stage [4]. Treatment typically aims to reduce pain, restore mobility, and enhance functional performance. Acute cases are treated with physiotherapy, pain medications, muscle relaxants, NSAIDs, intra-articular steroid injections, and hydrodilatation [5–7]. Chronic cases may require more invasive treatments such as manipulation under anesthesia (MUA) or surgical procedures, including capsular release and arthroscopic correction techniques [8,9]. Physiotherapy methods include thermotherapy, ultrasound, extracorporeal shock wave therapy (ESWT), interferential therapy (IFT), transcutaneous electrical nerve stimulation (TENS), manual therapy (MT), and therapeutic exercises [10,11]. However, while medical and surgical treatments can be effective, they often fail to address the underlying pathology and carry potential risks such as adverse drug reactions, tissue damage, and increased costs. Additionally, these interventions are not universally accepted by patients [12,13].

Despite the wide range of treatment options, the exact pathophysiology of frozen shoulder remains unclear, and there is no consensus regarding the best therapeutic approach. While intra-articular lidocaine (IAL) injections are commonly used, their clinical effectiveness in reducing inflammation, fibrous tissue formation, and inducing apoptosis remains unproven [14]. Furthermore, the use of IAL injections combined with physiotherapy, including manual therapy, has been suggested to enhance treatment outcomes. However, recent studies have shown inconsistent findings about the efficacy of this combination in reducing pain and improving functional outcomes in FS patients [15,16].

One of the significant gaps in research is the lack of studies evaluating both the clinical and radiological outcomes of intra-articular lidocaine injections combined with manual therapy in frozen shoulder. Although manual therapy (MT) has been proven effective in alleviating pain and improving the range of motion in FS patients, the impact of combining MT with intra-articular lidocaine injections on both clinical results and MRI findings is largely unexplored [17,18]. The lack of comprehensive evidence on this combination therapy highlights the need for further investigation [19,20].

This study was conducted to address the gap in knowledge regarding the combined effects of intra-articular lidocaine injections and manual therapy on frozen shoulder. The primary aim is to assess the clinical and MRI outcomes following this combined treatment approach. The proposed hypothesis suggests that there is no significant difference between the clinical and MRI outcomes of intra-articular lidocaine injections with manual therapy and those with sham manual therapy. By utilizing MRI to evaluate soft tissue changes in real-time, the study aims to provide valuable insights into the effectiveness of combining these treatments. The findings will offer evidence to guide clinicians in selecting the most effective treatment strategies for frozen shoulder, potentially improving patient outcomes and reducing the need for more invasive procedures.

## 2. Materials and methods

### 2.1 Study design

This randomized, single-blinded, parallel-group, sham-controlled trial was conducted at the Department of Physical Therapy and Health Rehabilitation, College of Applied Medical Sciences, Prince Sattam bin Abdulaziz University, Saudi Arabia. Participant recruitment took place from June 1, 2020, to August 31, 2023. The study adhered to the ethical principles outlined in the Declaration of Helsinki and received approval from the Department Ethics Committee (DEC), under approval number RHPT/020/014. The study declaration form was distributed to all the study participants and their consent to participant in the study was collected, in accordance with ethical guidelines. Additionally, the study was prospectively registered in a clinical trial registry with reference number CTRI/2020/04/024853 on April 25, 2020.

### 2.2 Participants

The study was carried out at the outpatient physiotherapy clinic of Prince Sattam bin Abdulaziz University, Saudi Arabia, the participants were referred from the University Hospital and King Khalid Hospital, Al-Kharj, Saudi Arabia. Screening for eligibility was performed by a senior orthopedic surgeon with over twenty years of experience in diagnosing and managing shoulder conditions. Eligible participants were aged 18–60 years, clinically diagnosed with frozen shoulder (International Classification of Diseases 10th revision [ICD-10]: M75.1–M75.8, M19.8), and reported pain severity in the range of 3 and 8 on the Visual Analogue Scale (VAS). Exclusion criteria included previous steroid injections, pain in the neck or arm region, glenohumeral osteoarthritis, severe neuromuscular conditions, psychosomatic disorders, patients awaiting surgeries, substance abuse, participation in weight training programs, upper limb fractures, soft tissue injuries, and deformities.

All participants received referral letters from their respective hospitals for rehabilitation and were provided with a pamphlet detailing the study’s procedures, benefits, and risks. A physiotherapist from the outpatient clinic explained the study protocol to participants, who were then invited to take part. Following the signing of informed consent and prior to baseline assessment, participants were randomly assigned to two groups utilizing a computer-generated random assignment method: the active group (n = 30) receiving intra-articular lidocaine injection with manual therapy and the sham group (n = 30) receiving lidocaine injection with sham manual therapy. Group allocation was managed by a separate physiotherapist using an on-site computer system to ensure concealment. Group assignments were disclosed to the intervening therapist performing manual therapy just before the first treatment, while participants remained blinded to their assigned treatment. As the nature of the interventions dictated, treating therapists were not blinded.

**Fig 1:**
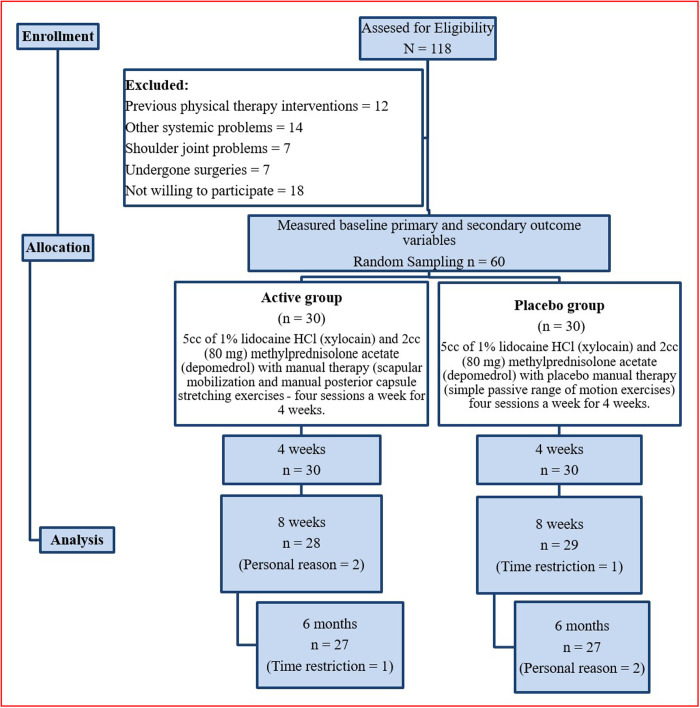
Flow of study participants in the active and sham groups.

Both groups received their respective interventions four times per week over four weeks. A blinded physical therapist assessed primary and secondary outcomes at baseline, after four weeks, eight weeks, and at six-month follow-up. Four physical therapists, with a minimum 15 years of expertise in shoulder rehabilitation, delivered the interventions. The participant flow throughout the study is depicted in Fig 1.

### 2.3 Interventions

Intra-articular lidocaine (IAL) injections were administered by two orthopedic surgeons, while four physical therapists provided the manual therapy interventions. After the IAL injection, participants underwent physiotherapy sessions for four weeks, followed by prescribed exercises at home for an additional four weeks. Each participant was given a handout with detailed exercise guidelines and was instructed to maintain an exercise logbook to document their training activities. A physiotherapist monitored participant adherence to the treatment regimen, rating it from ‘very good’ to ‘very poor.’

#### 2.3.1 Lidocaine injection.

Prior to the injection, the treating orthopedic surgeon performed a complete physical and orthopedic evaluation. Both groups received a posterior intra-articular injection using an 18-gauge spinal needle, delivering a mixture of 5cc of 1% Xylocaine and 2cc (80 mg) Depomedrol [21]. After the injection, participants were given printed brochures explaining post-injection care instructions, which were also explained verbally. Participants were advised to rest and refrain from vigorous activities for one week, regardless of pain relief, and to report any feedback on the injection to the treating surgeon. Any adverse effects were documented and managed by the primary investigator.

#### 2.3.2 Physiotherapy.

**Fig 2:**
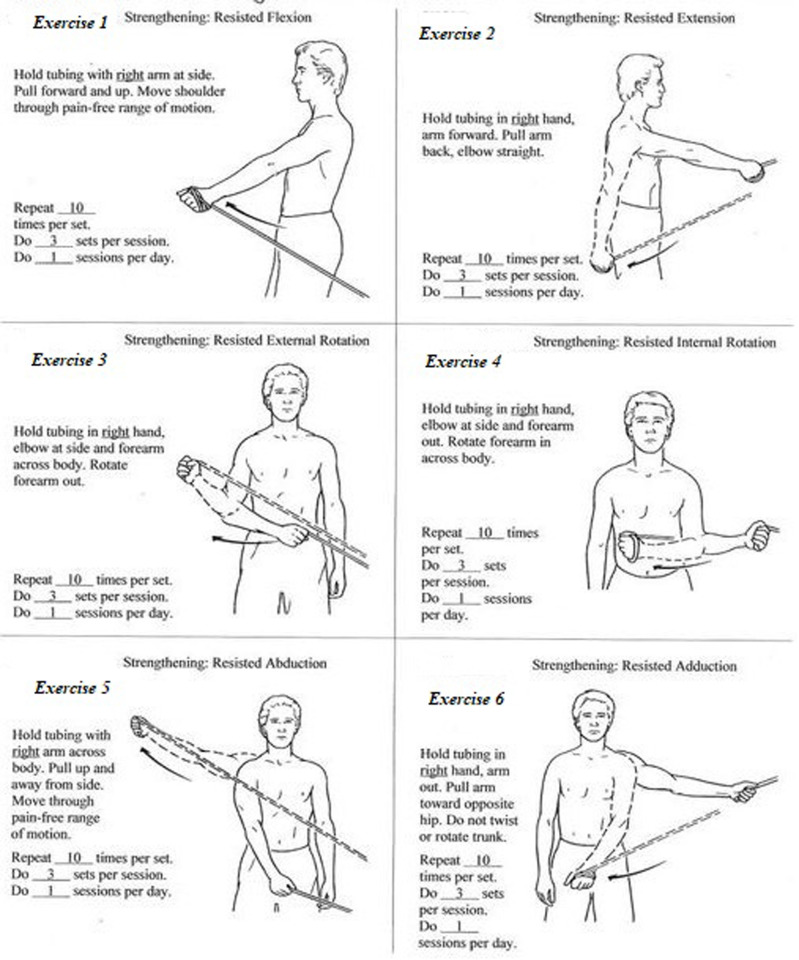
Fixed physiotherapy protocol (Appendix –A).

The shoulder rehabilitation protocols were based on previous evidence showing optimal outcomes. One week later, following the application of IUL injection, all the participants started physiotherapy under the supervision of a registered physiotherapist with over fifteen years of expertise in shoulder rehabilitation. Both active and sham groups attended four physiotherapy sessions a week for four weeks, each session lasting 30–40 minutes. A study by *Cacchio et al,* have shown that higher frequency of sessions (e.g., 3–5 sessions per week) for 4–6 weeks leads to more rapid improvements in pain reduction, range of motion (ROM), and function. The rationale behind this is that more frequent treatments may accelerate tissue remodeling and reduce pain more effectively. Therefore, the frequency of 4 sessions per week for 4 weeks can be considered optimal for many patients, especially when coupled with other therapeutic modalities, such as exercise and education. However, the “optimal” frequency and duration may vary depending on individual patient factors such as severity of the condition, stage of adhesive capsulitis, and the presence of comorbidities [22]. To minimize bias, a standardized physiotherapy protocol (Appendix A, Fig 2) was developed based on latest evidence, with aim of pain reduction, functional improvement, and soft tissue healing. The protocol combined physical modalities, exercise routines, and patient education.

#### 2.3.3 Manual therapy.

**Fig 3:**
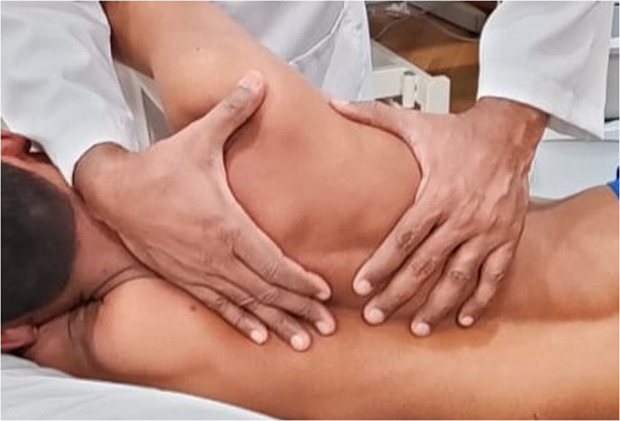
Application of scapular stabilization exercises.

Participants in the active intervention group received manual therapy techniques comprising scapular mobilization and posterior capsule stretching, performed by a licensed physiotherapist with expertise in musculoskeletal rehabilitation. Scapular mobilization was carried out with participants positioned in a lateral decubitus (side-lying) posture on the unaffected side, with the affected arm supported at 90 degrees of shoulder flexion in the scapular plane. The physiotherapist manually stabilized the scapula using one hand to prevent compensatory movement, while the other hand guided the scapula through controlled mobilization techniques. These included medio-lateral (side-to-side glides), supero-inferior (up-and-down glides), and circumduction (gentle circular movements), each performed ten times. A rest interval of 30 seconds was allowed between sets to avoid fatigue and allow for neuromuscular relaxation (Fig 3).

**Fig 4:**
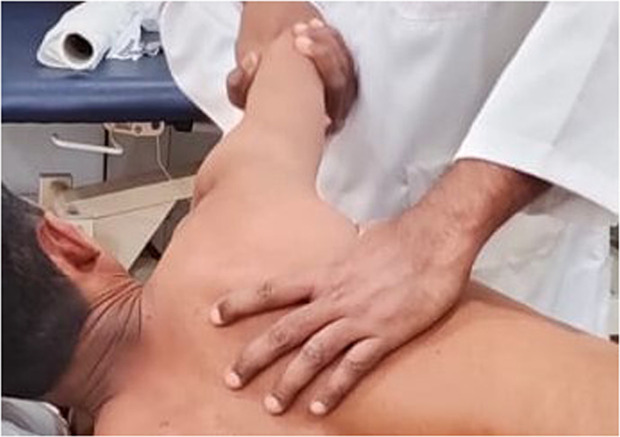
Application of posterior capsular stretching exercises.

Posterior capsule stretching was similarly conducted in the side-lying position with the affected shoulder on top. The scapula was stabilized laterally by the physiotherapist to isolate the glenohumeral joint. The arm was maintained at 90 degrees of flexion and gently horizontally adducted, while a sustained posteriorly directed force was applied at the elbow to stretch the posterior capsule of the shoulder joint. Each stretch was held for 20 seconds and repeated 10 times, with a 30-second rest between repetitions. This technique was specifically aimed at improving posterior shoulder mobility and reducing capsular tightness, which are common impairments associated with subacromial impingement. Throughout both interventions, care was taken to ensure patient comfort and avoid any provocation of pain, and all procedures were performed within the participant’s pain-free range of motion (Fig 4) [19].

In the sham group, the intervention closely resembled the real treatment without delivering therapeutic effects to ensure proper blinding and control for placebo responses. The sham intervention involved light touch or passive hand placement on the shoulder and upper arm without applying pressure, traction, or mobilization. Superficial stroking over the deltoid and the scapular region was used instead of deep tissue techniques, ensuring that the patient experienced hands-on interaction without actual mechanical effects. Simulated joint mobilization was performed by placing the hands in the correct positions but applying only minimal movement without reaching end-range mobilization or stretching the joint capsule. To enhance credibility, the therapist used neutral, reassuring verbal cues similar to those in the actual treatment, avoiding any suggestion of active therapeutic effects.

#### 2.3.4 Progressive resistance exercises (PRE).

Both the active and sham groups performed progressive resistance exercises (PRE) using a Thera tube (Theraband, Illinois, USA) to target the shoulder muscles. Initially, exercises were performed with minimal resistance to avoid exacerbating pain, progressively increasing resistance as joint movements improved. Later phases focused on rehabilitation specific to participants’ daily activities or functional needs. Exercise parameters—intensity, frequency, and duration—were tailored to each participant’s capacity. Proper form and posture were emphasized to promote healing. Participants also received a pamphlet with information about the condition and home exercises [23]. They were instructed to perform daily home exercises for four weeks, including eccentric exercises (3 sets of 30 repetitions) and isolated shoulder muscle stretches (3 times per day for 30 seconds). Therapist adherence was monitored by reviewing each participant’s exercise logbook at the start of every session.

### 2.4 Outcome measures

#### 2.4.1 Pain intensity.

The Visual Analogue Scale (VAS) was used to measure the pain levels, where participants recorded the pain level they experienced on a 10 cm scale ranging from “free from pain” (0) to “unmaginable pain” (10). VAS is a widely accepted and validated tool for evaluating pain in frozen shoulder patients [24].

#### 2.4.2 Magnetic resonance imaging (MRI).

MRI is a reliable and valid method for assessing coracohumeral ligament thickening in patients affected with FS. Scans were performed with a 3.0-T MR unit (Siemens Medical Solutions, Germany) and a phased-array surface coil positioned over the glenohumeral joint. The arm was positioned neutrally with the thumb pointing upwards. T1-weighted images were taken in the sagittal oblique plane parallel to the glenohumeral joint [25].

#### 2.4.3 Functional disability.

The Disabilities of the Arm, Shoulder, and Hand (Quick-DASH) questionnaire was utilized to evaluate physical impairment and symptoms of FS. It consists of 11 items with scores ranging from 0 (no impairment) to 100 (most severe disability), with demonstrated reliability and sensitivity to changes in shoulder-related disability [26].

#### 2.4.4 Kinesiophobia.

Fear of injury was assessed using the Tampa Scale for Kinesiophobia – Adjusted Version (TSK-AV), a 13-item scale rated on a 4-point Likert scale, where higher scores reflect greater fear of injury [27].

#### 2.4.5 Depression.

The Hospital Anxiety and Depression Scale (HADS) was employed to assess depression levels, with seven items for measuring depression and seven for anxiety. Each item is scored from 0 to 3, with scores above 8 indicating significant symptoms of depression or anxiety [28].

#### 2.4.6 Quality of life.

Health-related quality of life was evaluated using the EuroQol EQ-5D scale, generating utility values ranging from 1 (perfect health) to 3 [29].

### 2.5 Sample size

Based on the study by Bhatia DN et al [15], a minimum of 30 participants per group (total n = 60) was required to achieve a statistical power of 90% (β = 0.10) at a two-sided significance level of 0.05 (α = 0.05). This calculation was performed to detect a clinically meaningful difference of 4 points in the Visual Analog Scale (VAS) scores at the 6-month follow-up, assuming a standard deviation of 6 points. The estimated sample size without attrition was 24 participants per group; however, to account for an anticipated 25% dropout rate, the final sample size was adjusted to 30 participants per group. For secondary outcomes, a 20% difference was considered clinically significant.

### 2.6 Statistical analysis

Data analysis was performed by a statistician who was not involved in participant recruitment, evaluation, or treatment. The Kolmogorov-Smirnov test assessed the normality of data distribution by comparing the sample distribution of each variable against a reference normal distribution; a non-significant result (p > 0.05) indicated that the data were normally distributed and suitable for parametric analysis. The analysis followed the intention-to-treat principle, with missing data handled by using the most recent participant assessment. A Linear Mixed Model (LMM) was used to evaluate between-group differences in the effects of lidocaine injection with manual therapy, accounting for both fixed effects (treatment, time, and interactions) and random effects (individual variability). This model is ideal for analyzing repeated-measures data while handling missing values and intra-subject correlations. Additionally, Analysis of Variance (ANOVA) was used to compare the clinical outcomes across groups, assessing statistical differences in pain relief, MRI changes, functional disability, kinesiophobia status, depression level and quality of life. Together, these analyses provide a robust evaluation of treatment efficacy over time. Mean differences (MD) and 95% confidence intervals (CI) were calculated for each between-group comparison. Statistical analysis was conducted using IBM SPSS Statistics for Windows, Version 26.0 (Armonk, NY: IBM Corp), with a significance level set at p ≤ 0.05.

## 3. Results

### 3.1 Compliance with the trial protocol

The study successfully met the necessary sample size based on the pre-calculated minimum. Approximately 50% of screened participants fulfilled the eligibility criteria and were enrolled. All outcome measures specified in the registered protocol were assessed and reported, with no additional outcomes evaluated or documented.

### 3.2 Flow of participants

Initially, 118 participants were screened for eligibility. Twelve individuals had prior physical therapy, 14 had systemic health issues, seven had other shoulder conditions, seven had undergone joint surgeries, and 18 declined participations. As a result, 60 participants who met the inclusion criteria were enrolled, as shown in Fig 1. During the 6-month follow-up, three participants from both the active and sham groups withdrew for personal reasons and due to time constraints.

Baseline demographic and clinical data for the study participants are provided in Table 1. The average age ± standard deviation (SD) was 49.18 ± 4.2 years for the active group and 48.56 ± 4.1 years for the sham group, with no significant difference (p = 0.565). The gender distribution was equal, with 30 males (50%) and 30 females (50%) in each group. No significant differences were noted in height (p = 0.825) or weight (p = 0.797) between the groups. The data indicated that the dominant and right sides were more frequently affected in both groups. Regarding previous pain episodes, 15% (n = 9) of participants in both groups reported past pain experiences. The mean duration of pain was 6.3 ± 1.6 months in the active group and 6.5 ± 1.5 months in the sham group, with no significant difference (p = 0.619). In terms of employment, 76.66% (n = 46) were engaged in manual labor, 16.66% (n = 10) in non-manual jobs, and 6.66% (n = 4) were unemployed.

**Table 1:** Demographic and clinical characters of active and placebo groups.

Sr. No	Variable	Active (n = 30)	Sham (n = 30)	p-value
1	Age (y)	49.18 ± 4.2	48.56 ± 4.1	0.565
2	Gender			
	Male	13 (43%)	14 (47%)	–
	Female	17 (57%)	16 (53%)	–
3	Height (meter)	1.67 ± 0.18	1.66 ± 0.17	0.825
4	Weight (kg)	76.2 ± 6.1	75.8 ± 5.9	0.797
5	Side involved (%)			
	Right side	27 (90%)	28 (94%)	–
	Left side	2 (7%)	1 (3%)	–
	Bilateral	1 (3%)	1 (3%)	–
6	Dominance side (%)			
	Dominance = Right	28 (93%)	27 (90%)	–
	Dominance = Left	2 (7%)	3 (10%)	–
7	Previous episodes of pain N (%)	4/30 (17%)	5/30 (20%)	–
8	Duration of pain (months)	6.3 ± 1.6	6.5 ± 1.5	0.619
9	Employment			
	Manual work	22/30 (73%)	24/30 (80%)	–
	Non-manual work	6/30 (20%)	4/30 (13%)	–
	Not working	2/30 (7%)	2/30 (7%)	–

y – year, kg – kilogram.

### 3.3 Effects of the intervention

The linear mixed model (LMM) analysis of the primary variable (pain intensity measured by VAS) using a 4 × 2 time and group framework showed a statistically significant difference (p = 0.001) between the active and sham groups at baseline, 4 weeks, 8 weeks, and 6 months’ follow-up. At 4 weeks’ post-intervention, the active group demonstrated an improvement of 2.4 (95% CI: 2.08 to 2.71) compared to the sham group. Similar improvements were observed at 8 weeks (3.0, 95% CI: 2.68 to 3.31) and at 6 months (2.5, 95% CI: 2.18 to 2.81). These changes in scores were statistically significant (p < 0.001) in favor of the active group, as presented in Tables 2–4. The effect size for pain intensity (η² = 0.88) indicated a larger impact in the active group than the sham group.

**Table 2:** Pre and post primary and secondary outcome measures of active and placebo groups.

Sr. No	Variable	Duration	Active	Sham	Group × Time
1	Pain intensity – VAS (0–10 cm)	Base line	7.4 ± 1.4	7.3 ± 1.4	p = 0.001*
		4 weeks	3.2 ± 0.8	5.5 ± 1.2	
		8 weeks	1.4 ± 0.6	4.3 ± 0.9	
		6 months	0.8 ± 0.07	3.2 ± 0.6	
2	MRI T2 sagittal section (Thickness – mm)	Base line	4.4 ± 0.6	4.5 ± 0.5	p = 0.001*
		4 weeks	4.0 ± 0.5	4.4 ± 0.5	
		8 weeks	3.6 ± 0.4	4.2 ± 0.4	
		6 months	3.2 ± 0.2	4.0 ± 0.3	
3	Functional disability Q-DASH (0–100)	Base line	73.8 ± 7.1	73.5 ± 6.9	p = 0.001*
		4 weeks	36.2 ± 4.1	56.2 ± 5.2	
		8 weeks	20.5 ± 2.1	40.2 ± 4.5	
		6 months	9.8 ± 0.9	28.6 ± 3.1	
4	Kinesiophobia (TSK-AV)	Base line	48.2 ± 4.1	47.8 ± 4.0	p = 0.001*
		4 weeks	30.2 ± 3.2	38.5 ± 3.1	
		8 weeks	19.9 ± 2.7	29.2 ± 2.5	
		6 months	10.1 ± 1.6	19.2 ± 1.9	
5	Depression (HADS)	Base line	16.2 ± 1.5	16.4 ± 1.6	p = 0.001*
		4 weeks	12.8 ± 1.4	13.9 ± 1.4	
		8 weeks	8.1 ± 0.8	10.4 ± 0.9	
		6 months	4.3 ± 0.4	8.8 ± 0.8	
6	Quality of life (EuroQol EQ-5D)	Base line	2.7 ± 0.7	2.6 ± 0.7	p = 0.001*
		4 weeks	2.3 ± 0.6	2.4 ± 0.6	
		8 weeks	1.8 ± 0.4	2.3 ± 0.4	
		6 months	1.4 ± 0.2	2.2 ± 0.3	

*Significant, VAS – Visual analog scale, MRI – Magnetic resonance imaging, TSK – AV – The Tampa Scale for Kinesiophobia – adjusted version, HADS – Hospital Anxiety and Depression Scale, EuroQol EQ-5D – European quality of life – five dimension.

**Table 3:** Mean and (SD) difference within groups for primary and secondary outcome variables.

Variables	Difference within groups					
	Week 4 minus Baseline		Week 8 minus Baseline		Month 6 minus Baseline	
	Active	Sham	Active	Sham	Active	Sham
Pain intensity	−4.2 (0.5)	−1.8 (0.7)	−6.0 (0.5)	−3.0 (0.7)	−6.6 (0.5)	−4.1 (0.7)
MRI T2	−0.4 (0.3)	−0.1 (0.2)	−0.8 (0.3)	−0.3 (0.2)	−1.2 (0.3)	−0.5 (0.2)
Fun. Disability	−37.6 (2.8)	−17.3 (3.4)	−53.3 (2.4)	−33.3 (3.4)	−64.0 (2.4)	−44.9 (3.4)
Kinesiophobia	−18.0 (2.0)	−9.3 (2.0)	−28.3 (2.0)	−18.6 (2.0)	−38.1 (2.0)	−28.6 (2.0)
Depression	−3.4 (0.7)	−2.5 (0.8)	−8.1 (0.7)	−6.0 (0.8)	−11.9 (0.7)	−7.6 (0.8)
Quality of life	−0.4 (0.3)	−0.2 (0.3)	−0.9 (0.3)	−0.3 (0.3)	−1.3 (0.3)	−0.4 (0.3)

**Table 4:** Mean (95% CI upper – lower) difference between groups for primary and secondary outcome variables.

Variables	Difference between groups		
	Week 4 minus Baseline	Week 8 minus Baseline	Month 6 minus Baseline
	Active – Sham	Active – Sham	Active – Sham
Pain intensity	2.4 (2.08 to 2.71)	3.0 (2.68 to 3.31)	2.5 (2.18 to 2.81)
MRI T2	0.3 (0.16 to 0.43)	0.5 (0.36 to 0.63)	0.7 (0.56 to 0.83)
Fun. disability	20.3 (18.69 to 21.90)	20.0 (18.39 to 21.6)	19.1 (17.49 to 20.70)
Kinesiophobia	8.7 (7.66 to 9.73)	9.7 (8.66 to 10.73)	9.5 (8.46 to 10.53)
Depression	0.9 (0.51 to 1.28)	2.1 (1.71 to 2.48)	4.3 (3.91 to 4.68)
Quality of life	0.2 (0.04 to 0.35)	0.6 (0.44 to 0.75)	0.9 (0.74 to 1.05)

**Fig 5:**
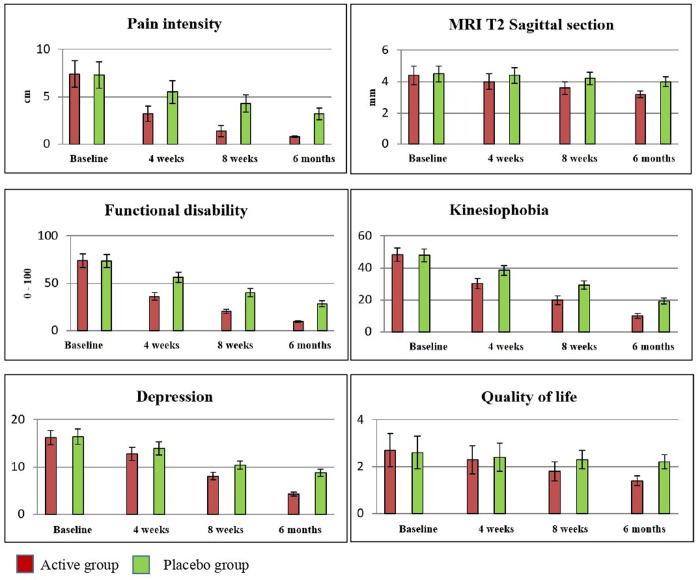
Pre and post outcome measures of active and sham groups.

For secondary outcomes, including MRI T2 sagittal section, functional disability, kinesiophobia, depression, and quality of life, the LMM analysis also demonstrated statistically significant differences (p = 0.001) between the groups at baseline, 4 weeks, 8 weeks, and 6 months follow-up. Post-intervention improvements in the active group at 4 weeks were as follows: MRI T2 sagittal section (0.3, 95% CI: 0.16 to 0.43), functional disability (20.3, 95% CI: 18.69 to 21.90), kinesiophobia (8.7, 95% CI: 7.66 to 9.73), depression (0.9, 95% CI: 0.51 to 1.28), and quality of life (0.2, 95% CI: 0.04 to 0.35). Similar improvements were observed at 8 weeks and 6 months follow-up: MRI T2 sagittal section (0.7, 95% CI: 0.56 to 0.83), functional disability (19.1, 95% CI: 17.49 to 20.70), kinesiophobia (9.5, 95% CI: 8.46 to 10.53), depression (4.3, 95% CI: 3.91 to 4.68), and quality of life (0.9, 95% CI: 0.74 to 1.05). Statistically significant improvements (p = 0.001) favoring the active group are outlined in Tables 2–4. The effect sizes for MRI T2 sagittal section (η² = 0.68), functional disability (η² = 0.78), kinesiophobia (η² = 0.84), depression (η² = 0.72), and quality of life (η² = 0.61) indicate stronger effects in the active group, as depicted in Fig 5.

#### 3.3.1 Adverse events with the study intervention.

No adverse reactions or side effects were reported in either the active or sham groups throughout the treatment and follow-up period.

## 4. Discussion

This randomized controlled trial investigated the potential benefits of manual therapy following intra-articular lidocaine (IAL) injection in patients with frozen shoulder (FS). The results demonstrated that participants in the active intervention group exhibited significantly greater improvements in both primary and secondary outcome measures compared to the sham group at various time points. However, the placebo group also showed statistically significant improvements over time when compared to baseline values. This suggests that FS may resolve naturally following an IAL injection without additional targeted treatment.

Rymaruk et al. reported that intra-articular lidocaine (IAL) injection significantly reduced pain severity and improved functional range of motion (ROM) in patients with frozen shoulder (FS) compared to those who did not receive the injection, emphasizing its effectiveness as a minimally invasive treatment option [30]. The mechanism of benefit is likely attributable to the stretching of the joint capsule via injection of 3 ml of 1% lidocaine, leading to mechanical distension and neurochemical pain modulation. Lidocaine, a commonly used local anesthetic, inhibits small nerve fibers, temporarily blocking both pain and autonomic signals, thus enabling early recovery through improved mobility [17].

Notably, IAL injection therapy has been favorably compared to more invasive interventions such as manipulation under anesthesia (MUA). MUA carries risks including humeral fracture, dislocation, and rotator cuff injury, and requires anesthesia support. In contrast, IAL injection is simpler, safer, cost-effective, and can yield rapid symptom relief [30]. However, concerns about chondrotoxicity have persisted. For instance, a clinical study linked continuous infusion of 0.5% bupivacaine via pain pump (377 ml total) to glenohumeral cartilage damage [31]. Nevertheless, in vitro research has indicated that a single administration of 1% lidocaine, as used in the current study, has a substantially lower chondrotoxic profile [31,32].

Hsu et al. demonstrated that IAL injections administered prior to physiotherapy sessions were more effective in pain relief and ROM improvement compared to physiotherapy alone [17]. Our findings are consistent with this, indicating superior outcomes when combining IAL with manual therapy. Importantly, early mobilization following pain relief can risk recurrence if not cautiously managed. Hence, patients in our study were instructed to restrict use of the affected limb for at least one-week post-injection to mitigate flare-ups.

Page et al., in a systematic review and meta-analysis, reported that joint distension combined with manual therapy and exercise interventions yielded significant improvements in pain, function, and quality of life among FS patients [33]. Similarly, Vermeulen et al. found that high-grade mobilizations, particularly anterior and posterior glides, were effective in improving external rotation and abduction [34]. In the present study, scapular mobilization techniques were employed to address biomechanical restrictions caused by capsular tightness and scapulothoracic dyskinesia—hallmarks of adhesive capsulitis. These techniques, along with posterior capsule stretches, targeted key structures limiting ROM, such as the coracohumeral ligament and posterior capsule [35].

While existing literature presents conflicting evidence regarding the additive benefits of manual therapy after IAL injection, this randomized controlled trial sought to clarify this gap. The results demonstrated that manual therapy significantly contributed to pain reduction, enhanced ROM, and functional improvement, possibly due to mechanical and neurophysiological effects. Manual therapy disrupts intra-articular adhesions, reorganizes collagen fibers, and improves joint extensibility [36]. The “Pain Gate Theory” further elucidates its analgesic effect, proposing that joint mobilization stimulates large-diameter afferent fibers, which inhibit nociceptive transmission at the spinal level [37].

Exercises focusing on scapular mobilization and posterior capsular stretching aid in improving shoulder movement, alleviating tissue stiffness, and relaxing muscles due to the sedative effects of joint manipulation. Collectively, these factors contribute to reduced pain intensity, improved ROM, and enhanced joint functionality [38]. Our findings further reinforce that incorporating manual therapy alongside progressive resistance exercises optimizes recovery. Furthermore, interventions targeting scapular mechanics and posterior capsule extensibility also likely contributed to pain modulation and functional gains. Cools et al. and Borstad et al. emphasized the importance of restoring scapular kinematics in shoulder rehabilitation, noting improved glenohumeral coordination and decreased subacromial impingement when scapular mobility is enhanced [39,40].

MRI-based assessment of coracohumeral ligament (CHL) thickening—used in our study as an objective diagnostic marker—is supported by Mengiardi et al., who found CHL hypertrophy to correlate strongly with limitations in external rotation and pain levels in FS patients [41]. The use of sagittal imaging allowed precise localization and thickness quantification, aiding in diagnosis and monitoring. Our study further demonstrated sustained improvements in Q-DASH scores across all follow-ups, indicating robust functional recovery [23,42]. These findings align with those of Lee SY et al., who reported that combining physical therapy with pain control measures improved not only physical outcomes but also psychological well-being by reducing fear-avoidance behaviors and depression in FS patients [43].

### 4.1 Implications of findings

The synergistic combination of IAL injection and manual therapy, complemented by progressive resistance exercises, appears to offer a multimodal benefit in the management of FS. This protocol supports rapid symptom resolution, improves joint biomechanics, and enhances both physical and psychological recovery, potentially reducing chronicity. Given the multifactorial nature of FS, a multimodal, staged rehabilitation model seems most effective.

#### 4.1.1 Strengths of the study.

This randomized controlled trial demonstrated several methodological strengths while evaluating the effectiveness of various manual therapy interventions over 8 weeks and 6 months. One of the key strengths was the use of standardized physiotherapy techniques within a controlled clinical setting, which enhances the external validity and real-world applicability of the findings. The study strictly adhered to CONSORT guidelines, incorporating robust methods such as randomization, allocation concealment, and blinding to minimize bias. High participant retention and strong adherence to the intervention protocol further strengthened the reliability of the results. Additionally, appropriate statistical analyses were used to control for Type I errors, with careful consideration of the condition’s typically self-limiting and non-serious nature. These methodological strengths contribute to the credibility and clinical relevance of the study outcomes.

#### 4.1.2 Limitations of the study.

First, the relatively small sample size and single-center design may limit the generalizability of the findings to the broader population of patients with frozen shoulder. Second, this study followed the rigid physiotherapy protocol, which restricted individualized treatment adjustments and may have influenced the outcomes. Third, the lack of control group which consists of only manual therapy intervention which would have provided further insight into its isolated effects. Fourth, the reliance on subjective pain assessment using the Visual Analogue Scale (VAS), which may introduce variability due to differences in patients’ self-reporting. Additionally, while MRI was used to measure coracohumeral ligament thickness, this imaging parameter may not comprehensively capture all soft-tissue changes associated with frozen shoulder, potentially limiting the full evaluation of pathological changes. Nonetheless, we acknowledge the significance of this aspect and suggest it as a valuable direction for future research to better elucidate the independent role of manual therapy in the management of frozen shoulder. Furthermore, given the extensive existing literature on manual therapy alone, future studies should focus on evaluating its effectiveness in acute and sub-acute stages of the condition using comparable inclusion criteria to our study. In future studies, increase the sample size and multicenter recruitment would enhance the generalizability of the results and provide more reliable evidence for the treatment’s effectiveness in a broader patient population. Also, include a group that receives only manual therapy would help to determine the independent effects of manual therapy on frozen shoulder.

## 5. Conclusion

In conclusion, the combined approach of intra-articular lidocaine injection, manual therapy, and progressive resistance exercises effectively reduces pain, enhances functional capacity, alleviates kinesiophobia and depressive symptoms, and improves health-related quality of life in individuals with frozen shoulder.

## Supporting information

S1 File Study protocol. (DOCX)

S2 File CONSORT 2010 checklist of information to include when reporting a randomised trial*. (RTF)
