# Supplementary material for: Efficacy of MRI and clinical findings of Lidocaine injection combined with manual therapy in frozen shoulder—A prospective, randomized, single-blinded, sham-controlled trial
Source: PLoS One. 2025 Aug 6;20(8):e0328783. doi: 10.1371/journal.pone.0328783 (PMC12327625; doi:10.1371/journal.pone.0328783)
Supplement: S1 File — (DOCX) [file pone.0328783.s001.docx]

**Title:** Efficacy of MRI and clinical findings of Lidocaine injection combined with manual therapy in Frozen shoulder – A prospective, randomized, double-blinded, placebo-controlled trial.

Gopal Nambi,^1^ Mshari Alghadier,^1^ Elturabi Elsayed Ebrahim,^2^ Mudathir Mohamedahmed Eltayeb ^2^, Dena Eltabey Sobeh,^2^ Osama R. Aldhafian,^3^ Shahul Hameed Pakkir Mohamed,^4,5^ Naif A. Alshahrani,^6^ Faizan Kashoo,^7^ Hariraja Muthusamy,^7,8^ Radhakrishnan Unnikrishnan,^7,8^ Alaa Jameel A. Albarakati^9^

**^1^** Department of Physical Therapy and Health Rehabilitation, College of Applied Medical Sciences, Prince Sattam bin Abdulaziz University, Al Kharj, Saudi Arabia.

**^2^** Department of Medical & Surgical Nursing, College of Nursing, Prince Sattam bin Abdulaziz University, Al Kharj, Saudi Arabia.

^3^ Department of Surgery, College of Medicine, Prince Sattam bin Abdulaziz University, Al Kharj, Saudi Arabia.

^4^ Department of Physical therapy, Faculty of Applied Medical Sciences, University of Tabuk, Tabuk, Saudi Arabia.

^5^ Adjunct Professor, Saveetha College of Physiotherapy, Saveetha Institute of Medical and Technical Sciences, (Deemed to the University), Chennai 600077, TamilNadu, India.

^6^ Orthopedic Surgery Department, King Fahad Medical City, Ministry of Health, Riyadh 12231, Saudi Arabia.

**^7^** Department of Physical Therapy and Health Rehabilitation, College of Applied Medical Sciences, Majmaah University, Majmaah, Saudi Arabia.

^8^ PhD Scholar, Saveetha Institute of Medical and Technical Sciences, Chennai, India.

^9^ Department of Surgery, College of Medicine, Umm Al-Qura University, Al-Qunfudah Branch, Makkah, Saudi Arabia.

**Ethics approval and consent to participate:** This study was designed and conducted in accordance with the principles of the Declaration of Helsinki and it has been approved by the Department Ethics Committee, Prince Sattam bin Abdulaziz University, Saudi Arabia with an ethical approval number RHPT/020/014. The participants were informed about the harms and benefits of the research through an information form. Subjects who consented to participate in the study were selected for the study and the written **informed consent was obtained from all subjects.**

**Clinical trial registration:** Registered in Clinical trial registry, India with registration number CTRI/2020/04/024853 and registered prospectively on 25/04/2020. [https://ctri.nic.in/Clinicaltrials](https://ctri.nic.in/Clinicaltrials/rmaindet.php?trialid=42910&EncHid=12150.14677&modid=1&compid=19)

**Study Protocol**

**Background:** There are wide number of evidences supporting the use of different interventions for frozen shoulder. Recent studies have shown that magnetic resonance imaging (MRI) is used to find the thickening of the coracohumeral ligament (CHL) and joint capsule, and the obliteration of fat under the coracoid process. Therefore, along with the regular investigation procedures, there is a need to find the MRI changes after lidocaine injection with manual therapy in frozen shoulder. So far no studies have been conducted to find the radiological and clinical changes after lidocaine injection with manual therapy in treating frozen shoulder.

**Objective:** The objective of this study is to investigate the clinical and magnetic resonance image (MRI) changes after lidocaine injection with manual therapy in frozen shoulder.

**Design:** This trial will be a prospectively registered, randomized, single-blinded, parallel-group, sham-controlled trial conducted at the department of physical therapy and health rehabilitation, college of applied medical sciences, Prince Sattam bin Abdulaziz University, Al Kharj, Saudi Arabia. The participants will be recruited between 1^st^ June 2020 and 31^st^ August 2023. This study will be designed and conducted in accordance with the principles of the Declaration of Helsinki and it has been approved by the Department ethics committee (DEC) with an ethical approval number RHPT/020/014. A written informed consent will be obtained from all the study participants as per the ethical guidelines. The study was registered prospectively in a trial registry with reference number: CTRI/2020/04/024853 on 25/04/2020.

***Subjects***

The study will be conducted in outpatient physiotherapy clinic, Prince Sattam bin Abdulaziz University, Saudi Arabia and they will be referred from University hospital and King Khalid hospital, Al-Kharj, Saudi Arabia. An orthopedic surgeon with twenty years of clinical experience in diagnosing and treating shoulder conditions will be screened the participants to include in the study. The participants between 18 and 60 years of age with a clinical diagnosis of frozen shoulder (International Classiﬁcation of Diseases 10th revision [ICD-10] group M75.1-M75.8, M19.8) and the pain intensity 3 to 8 on the visual analogue scale (VAS) will be allowed to participate in the study. Participants with prior steroid injection therapy, associated neck or arm pain, glenohumeral osteoarthritis, severe musculoskeletal, neural, somatic and psychiatric problems, waiting for any surgeries, having alcohol or drug abuse, involving in any weight training programs will be excluded from the study. In addition, participants with other soft tissue injuries, fracture at the upper limb, and deformities will also be excluded from the study.

***Intervention***

The intra-articular lidocaine (IAL) injection will be given by two orthopedic surgeons and the manual therapy was given by four physical therapists. Following the IAL injection, the recommended physiotherapy will be given for 4 weeks, after which the participants will be asked to do the prescribed exercises at home for another 4 weeks. This will be executed by providing the patient with a hand-out which included instructions regarding do’s and don’ts while performing these exercises. They will be asked to keep an exercise log book to enter their training activities during the study period. A physiotherapist continuously monitored all the participants involved in the study and graded them for adherence to the treatment from ‘very good’ to ‘very poor’.

*Lidocaine injection*

An orthopedic surgeon did a regular physical and orthopedic examination before the administration of the injection. Both the groups will be treated with a posterior approach intra-articular injection containing a mixture of 5cc of 1% lidocaine HCl (Xylocaine) and 2cc (80 mg) methylprednisolone acetate (Depomedrol) via an 18-gauge spinal needle. In addition, post-injection instructions will be given to all the participants by providing a printed brochure and also explaining it to them personally. They will be asked to take rest and not engage in strenuous activities for one week following the injection, even if they experience pain relief. They will also ask to inform about the feedback and response of injection to the concerned treating surgeon. Any adverse consequences will be noted and treated by the primary investigator.

*Physiotherapy*

The shoulder physiotherapy rehabilitation protocols will be prescribed based on the previous evidence that has shown the best results. After one week, following injection therapy, all the participants will be allowed to take regular physiotherapy interventions by a licensed physiotherapist with fifteen years of clinical experience in treating shoulder conditions. Participants in both groups will receive physical therapy treatment for 4 sessions per week for 4 weeks and each session lasted for 30 to 40 minutes.

All the participants will undergo an initial baseline evaluation of variables before the administration of the injection. To avoid intervention bias, a fixed physiotherapy protocol (Appendix –A, fig 2) will be prepared on the basis of recent evidence with the objectives of ameliorating pain, increasing functional activities and soft tissue healing. A holistic approach consisting of physical modalities, exercise protocols and patient education will be used to obtain these objectives.

Manual therapy: Participants in the active group will receive the manual therapy in the form of scapular mobilization and manual posterior capsule stretching exercises. Scapula mobilization will be performed with patients lying on their sides with their arms at 90^O^ flexion. The physiotherapist held the scapula from the medial border and applied medio-lateral, supero-inferior, and circumduction movements 10 times each. A 30 s break will be given between each practice (fig 3A). Posterior capsule stretching will be applied with patients lying in a lateral position. The scapula will be stabilized at the lateral side with the arm was at 90^o^ flexion. Stretching will be applied from the elbow with a downward force. The stretch will be repeated 10 times for 20 seconds each. A 30 seconds break will be given between each stretching (fig 3B).^19^ For the placebo group, simple passive range of motion exercises will be performed at the shoulder joint, which will not comprehensible to the study participants.

Progressive resistance exercises (PRE): Participants in both active and placebo groups will be prescribed with PRE exercises (Appendix A) with Thera tube (Theraband, Illinois, USA) for the shoulder muscles based on the assessment of individual muscles. In the early phase, the painful movements will be trained with minimal resistance and then progressed to the next level of resistance for the other joint movements. In the later phase, the progression of exercise intends to work on activity or function-specific rehabilitation. The therapist will select the exercise parameters (intensity, frequency and duration) in every treatment session purely based on the individual capacities without exaggerating the symptoms. Throughout the treatment session, the participants will be instructed to follow the correct form and posture to facilitate healing. Patient guidance will be given through patient counselling on an individual basis and a pamphlet regarding the disease and home instructions were given to all the participants. The patients will perform the home exercises daily for four weeks with eccentric exercise (3 times 30 repetitions) and isolated stretching of shoulder muscles (3 times daily for 30 seconds). The treatment adherence at home will be monitored by a treating therapist before the commencement of every session by checking the exercise log book.

***Outcome measures***

*Pain intensity:* It will be measured with visual analogue scale (VAS) and the participant will be asked to note the perceived pain intensity on the 10 cm point scale, where scores ranged from 'no pain' (0) to 'worst imaginable pain' (10). VAS is considered a valid and reliable tool for measuring pain intensity in frozen shoulder patients.^23^

*Magnetic resonance imaging (MRI):* It has been established as a reliable and valid assessment tool to measure the thickening of corocohumeral ligament in frozen shoulder patients. It will be performed with a 3.0-T MR unit (Siemens Medical Solutions, Germany) with a phased-array surface coil (Philips, Nederland) centred over the glenohumeral joint and strapped in place. The arm position was standardized, with the thumb pointing upward in a neutral position. In the sagittal oblique plane, parallel to the glenohumeral joint (550/15, 3-mm section thickness, 0.3-mm intersection gap, 1806180-mm field of view, 5126512 matrix size) T1 weighted sections were taken.^24^

*Functional disability:* The Disabilities of the Arm, Shoulder and Hand questionnaire (Quick- DASH), will be used to measure the upper limb physical disabilities and symptoms in frozen shoulder. It contains 11 items summarized into a total score from 0 ‘no disability’ to 100 ‘most severe disability. The Quick-DASH has adequate reliability, validity and ability to measure changes in disability among people with shoulder problems.^25^

*Kinesiophobia:* The Tampa Scale for Kinesiophobia – adjusted version (TSK-AV) was used to measure the status of fear of injury. The scale consists of 13 items, which are marked on a 4-point Likert scale. Getting a maximum score indicates more fear of injury and less score indicates less fear of injury.^26^

*Depression:* The Hospital Anxiety and Depression Scale (HADS) will be used to measure the depression status of frozen shoulder patients. It consists of seven items each for depression and anxiety subscales. Scoring for each item ranges from 0 to 3, with 3 denoting the highest anxiety or depression level. A total subscale score of >8 points out of a possible 21 denotes considerable symptoms of anxiety or depression.^27^

*Quality of life:* The EuroQol EQ-5D will be used to measure the health-related quality of life, expressed as utility values ranging from 1 to 3, where 1 represents perfect health.^28^

**Statistical analysis plan**

With a power of 0.8 and a significance level of 0.05, at least 30 participants were needed to be included in each treatment arm (60 participants in total) to detect a clinically important mean difference between groups of 4 points on the VAS scores at 6 months’ follow-up period, when assuming a standard deviation of 1 point and considering a 10% drop to follow-up. For other outcomes, we considered a between-group difference of 20% of the outcome measure’s scale to be clinically worthwhile.

The data analysis will be performed by a statistician who did not participate in the recruitment, evaluation and treatment aspects of the study. The study homogeneity will be analyzed through the Kolmogorov-Smirnov test. The data analysis will be performed on an intention-to-treat principle. For the missing data, results obtained in the last available assessment of each participant were repeated. Analysis of variance with a linear mixed model (LMM) will be used to compare the effects of lidocaine injection with active versus placebo groups. The mean difference (MD) and 95% confidence interval (CI) will also be calculated for each between-group comparison. The statistical analyses will be processed using commercial statistical software (IBM SPSS Statistics for Windows, Version 26.0. Armonk, NY: IBM Corp) and a level of significance of p≤0.05 was adopted for all tests.

**Informed Consent Form**

Name of patient: Age/Gender:

**Study Title:** “Efficacy of MRI and clinical findings of Lidocaine injection combined with manual therapy in Frozen shoulder – A prospective, randomized, double-blinded, placebo-controlled trial.”

**Introduction and Purpose of Study:** You have been selected to take part in a study “Efficacy of MRI and clinical findings of Lidocaine injection combined with manual therapy in Frozen shoulder – A prospective, randomized, double-blinded, placebo-controlled trial.”

**Study Information**

**Outline of Procedures:** At first consultation you will be screened and evaluated for suitability of the study. You are requested to attend 4 sessions a week for 8 weeks’ period. If you are taking any medication or undergoing any other form of treatment for your pain, you may be excluded from the study.

**Risks and Discomfort:** The treatment is safe and is unlikely to cause any adverse side effects. All treatments will be performed by qualified physiotherapist.

**Benefits of the study:** This study will assist the physiotherapy profession in expanding its knowledge of this condition and thus making future treatment of patients suffering from chronic low back pain more effective.

**Withdrawal from the Study:** You are free to withdraw at any stage with no negative repercussions to your health care.

**Remuneration:** Patients taking part in the study will not be offered any other form of remuneration for taking part in the study.

**Costs of Study:** Treatment for the duration of the research process will be free of charge. **Confidentiality:** All patient information and results will be kept confidential and can be shared for research purpose if required.

I have been explained about this research in which I agreed to participate. I know that I am giving this consent without any force. I can discontinue the study any time without any reason and that will not affect my treatment that I have been informed. My identity can be disclosed for any other follow up research.

**Signature of Patient Signature of Researcher**

I certify that I have explained to the participant about nature, purpose, potential benefits & possible risk of the indicated procedure. The information collected will be kept confidential.

**Signature of Witness Signature of Researcher**

**DECLARATIONS**

**Ethics approval and consent to participate:** This study was designed and conducted in accordance with the principles of the Declaration of Helsinki and it has been approved by the Department Ethics Committee, Prince Sattam bin Abdulaziz University, Saudi Arabia with an ethical approval number RHPT/020/014. The participants were informed about the harms and benefits of the research through an information form. Subjects who consented to participate in the study were selected for the study and the written **informed consent was obtained from all subjects.**

**Clinical trial registration:** Registered in Clinical trial registry, India with registration number CTRI/2020/04/024853 and registered prospectively on 25/04/2020. [https://ctri.nic.in/Clinicaltrials](https://ctri.nic.in/Clinicaltrials/rmaindet.php?trialid=42910&EncHid=12150.14677&modid=1&compid=19)

**Consent for publication:** No individual data is contained in this publication.

**Availability of data and materials:** Data is available.

**Competing interest:** None declared

**Funding:** This study is supported via funding from Prince Sattam bin Abdulaziz University project number (PSAU/2023/R/1444).

**Acknowledgement**: This study is supported via funding from Prince Sattam bin Abdulaziz University project number (PSAU/2023/R/1444).
