# Supplementary material for: Efficacy of MRI and clinical findings of Lidocaine injection combined with manual therapy in frozen shoulder—A prospective, randomized, single-blinded, sham-controlled trial
Source: PLoS One. 2025 Aug 6;20(8):e0328783. doi: 10.1371/journal.pone.0328783 (PMC12327625; doi:10.1371/journal.pone.0328783)
Supplement: S2 File — (RTF) [file pone.0328783.s002.rtf]

CONSORT 2010 checklist of information to include when reporting a randomised trial*

			Item				Reported			
	Section/Topic		No			Checklist item	on page No			
	Title and abstract									
			1a		Identification as a randomised trial in the title	Page: 1 
Line: 2 			
			1b		Structured summary of trial design, methods, results, and conclusions (for specific guidance see CONSORT for abstracts)	Page: 3
Line: 4 - 25  			
	Introduction									
	Background and		2a		Scientific background and explanation of rationale	Page: 5
Line: 16 - 20			
	Objectives		2b		Specific objectives or hypotheses	Page: 5
Line: 29 - 30			
	Methods									
										
	Trial design		3a		Description of trial design (such as parallel, factorial) including allocation ratio	Page: 6
Line: 10 -16			
			3b		Important changes to methods after trial commencement (such as eligibility criteria), with reasons	Page: 6
Line: 17 – 18			
	Participants		4a		Eligibility criteria for participants	Page: 6
Line: 24 – 30			
			4b		Settings and locations where the data were collected	Page: 6
Line: 19 – 23			
	Interventions	5			The interventions for each group with sufficient details to allow replication, including how and when they were
actually administered	
Page:  7 - 8
Line: 19 – 31
1 - 31			
										
	Outcomes		6a		Completely defined pre-specified primary and secondary outcome measures, including how and when they				
						were assessed	Page: 9
Line: 4 – 24			
			6b		Any changes to trial outcomes after the trial commenced, with reasons	Page: 9
Line: 4 – 24			
	Sample size		7a		How sample size was determined	Page: 9
Line: 25 – 30			
			7b		When applicable, explanation of any interim analyses and stopping guidelines	Page: 9
Line: 30			
	Randomisation:									
	Sequence		8a		Method used to generate the random allocation sequence	Page: 7
Line: 1 – 12			
	Generation		8b		Type of randomisation; details of any restriction (such as blocking and block size)	Page: 7
Line: 1 – 12			
	Allocation	9			Mechanism used to implement the random allocation sequence (such as sequentially numbered containers),				
	concealment					describing any steps taken to conceal the sequence until interventions were assigned				
	Mechanism						Page: 7
Line: 1 – 12			
	Implementation	10			Who generated the random allocation sequence, who enrolled participants, and who assigned participants to				
						Interventions	Page: 7
Line: 1- 12			
	Blinding		11a		If done, who was blinded after assignment to interventions (for example, participants, care providers, those	Page:7
Line: 1 – 12			
										
	CONSORT 2010 checklist						Page 1		

		assessing outcomes) and how		
	11b	If relevant, description of the similarity of interventions		
Statistical methods	12a	Statistical methods used to compare groups for primary and secondary outcomes	Page: 10
Line: 1 – 19	
	12b	Methods for additional analyses, such as subgroup analyses and adjusted analyses	
Results				
Participant flow (a	13a	For each group, the numbers of participants who were randomly assigned, received intended treatment, and	
diagram is strongly		were analysed for the primary outcome	Page: 10
Line: 18 -23	
recommended)	13b	For each group, losses and exclusions after randomisation, together with reasons	Page: 10
Line: 24 - 30	
Recruitment	14a	Dates defining the periods of recruitment and follow-up	Page: 10
Line: 24 – 30	
	14b	Why the trial ended or was stopped	Page: 10
Line: 18 -23	
Baseline data	15	A table showing baseline demographic and clinical characteristics for each group	Page: 10
Line: 24 – 30	
Numbers analysed	16	For each group, number of participants (denominator) included in each analysis and whether the analysis was	
		by original assigned groups	Page: 9
Line: 24 – 30	
Outcomes and	17a	For each primary and secondary outcome, results for each group, and the estimated effect size and its	
Estimation		precision (such as 95% confidence interval)	Page: 10
Line: 18 -23	
	17b	For binary outcomes, presentation of both absolute and relative effect sizes is recommended	Page: 10
Line: 24 – 30	
Ancillary analyses	18	Results of any other analyses performed, including subgroup analyses and adjusted analyses, distinguishing	
		pre-specified from exploratory	Page: 10
Line: 18 -23	
Harms	19	All important harms or unintended effects in each group (for specific guidance see CONSORT for harms)	
Discussion				
Limitations	20	Trial limitations, addressing sources of potential bias, imprecision, and, if relevant, multiplicity of analyses	Page: 12
Line: 1 – 30	
Generalisability	21	Generalisability (external validity, applicability) of the trial findings	Page: 12
Line: 1 -30	
Interpretation	22	Interpretation consistent with results, balancing benefits and harms, and considering other relevant evidence 	
Other information				
Registration	23	Registration number and name of trial registry	Page: 6
Line: 17 – 18	
Protocol	24	Where the full trial protocol can be accessed, if available	Page: 2
Line: 12 - 13	
Funding	25	Sources of funding and other support (such as supply of drugs), role of funders                                                    Page 2; 
                                                                                                                                                                                  line: 14-15	
			

*We strongly recommend reading this statement in conjunction with the CONSORT 2010 Explanation and Elaboration for important clarifications on all the items. If relevant, we also recommend reading CONSORT extensions for cluster randomised trials, non-inferiority and equivalence trials, non-pharmacological treatments, herbal interventions, and pragmatic trials. Additional extensions are forthcoming: for those and for up to date references relevant to this checklist, see www.consort-statement.org.


CONSORT 2010 checklist	Page 2
